# Edlan-Mejchar Procedure: Enhancing Complete Denture Retention

**DOI:** 10.7759/cureus.78346

**Published:** 2025-02-01

**Authors:** Rajani Patil, Girish Suragimath, Siddhartha Varma, Sameer A Zope, Ashwinirani SR

**Affiliations:** 1 Department of Periodontology, School of Dental Sciences, Krishna Vishwa Vidyapeeth (Deemed to be University), Karad, IND; 2 Department of Oral Medicine and Radiology, School of Dental Sciences, Krishna Vishwa Vidyapeeth (Deemed to be University), Karad, IND

**Keywords:** complete denture retention, edlan-mejchar, keratinized mucosa, pre-prosthetic surgery, vestibuloplasty

## Abstract

The retention, stability, and function of a complete denture require the presence of adequate width of attached keratinized tissue and vestibular depth. Pre-prosthetic surgical procedures are performed to fulfil these requirements for long-term success. The Edlan-Mejchar vestibuloplasty technique addresses challenges posed by mandibular ridge atrophy and insufficient vestibular depth in edentulous patients. This case report details the successful application of the Edlan-Mejchar procedure in a 58-year-old female patient referred for vestibular deepening to facilitate complete denture adaptation and retention. The preoperative evaluation revealed a severely resorbed mandibular ridge with a shallow vestibule. A split-thickness mucosal flap was repositioned inferiorly at the base of the vestibule, and the raised periosteum was sutured onto the lip mucosa to enhance vestibular depth and keratinized mucosa. Postoperative care included antibiotics, anti-inflammatory medications, and dietary modifications, with follow-up showing improved vestibular depth. The technique highlights the importance of adequate vestibular depth for functional denture retention. The Edlan-Mejchar procedure is effective in increasing keratinized mucosa and enhancing denture stability and patient comfort. The limitations of this procedure include the technique-sensitive nature of the surgery and its application in patients with systemic conditions affecting wound healing. This case report reaffirms the Edlan-Mejchar technique as a reliable, functional, and aesthetic solution for addressing soft tissue deficiencies in edentulous patients. We achieved a 6 mm gain in the keratinized attached mucosa and a deepened vestibule, which will ultimately help in denture stability and retention.

## Introduction

Teeth are needed for biting and chewing food to maintain good nutrition and overall health. Therefore, it is important to replace missing or lost teeth. There are several ways to replace missing teeth, including fixed prostheses, such as dental implants and bridges, and removable prostheses, such as partial and complete dentures. Fixed prostheses are popular for their stability and long-term success. However, they may not be suitable for completely edentulous patients with severe ridge resorption due to anatomical, financial, or systemic limitations. Complete dentures are the primary method of rehabilitation for full-mouth edentulism in such compromised cases.

The hard and soft tissues of the mandible undergo various changes due to ageing and loss of teeth. The hard tissue changes observed are progressive alveolar bone resorption, especially in edentulous patients, and a reduction in bone height and width, particularly in the anterior mandible. Cortical bone becomes sclerotic and brittle due to changes in trabecular pattern affecting load-bearing capacity. The soft tissue changes are loss of attached gingiva and mucosal thinning, decreased elasticity, and increased fibrosis of the mucosa. Migration of muscle attachment closer to the ridge crest results in reduced vestibular depth and alveolar ridge flattening, leading to compromised denture retention. A resorbed residual ridge and shallow vestibular depth can impact denture stability, often caused by prolonged denture use or advanced periodontal disease. Seibert, in 1983, classified ridge defects as class I: buccolingual loss of tissue (width deficiency), class II: apicocoronal loss of tissue (height deficiency), and class III: combination of both (severe resorption). We can address these challenges through pre-prosthetic surgeries for better rehabilitation outcomes.

"Pre-prosthetic surgery" refers to procedures performed to prepare the hard or soft tissues for prosthetic rehabilitation. The removal of tissue interferences, such as bony spikes, mylohyoid ridge, genial tubercle, hyperplastic tuberosity, exostosis, hyperplastic, hypertrophic, and hypermobile soft tissue, is recognized as pre-prosthetic and reconstructive surgeries [1].

Vestibuloplasty is a pre-prosthetic surgical procedure that corrects the inadequate keratinized attached tissue of the alveolar ridge [2]. The vestibuloplasty procedure is performed on patients with inadequate keratinized attached mucosa, which is a prerequisite for complete denture retention and stability. Various techniques for vestibuloplasty include submucosal, secondary epithelialization, Edlan-Mejchar methods, and soft tissue grafting. The Edlan-Mejchar method is a vestibular deepening procedure that was introduced by Edlan and Mejchar in 1963 [3]. The advantage of the Edlan-Mejchar procedure is achieving predictable vestibular depth without the use of soft tissue graft from the donor site, preventing the donor site morbidity. This case report highlights the successful use of Edlan-Mejchar vestibuloplasty to deepen the vestibule and increase the keratinized attached mucosa in the mandibular anterior region, ensuring improved prosthetic retention and stability of complete denture in an edentulous patient [4].

## Case presentation

**Figure 1:**
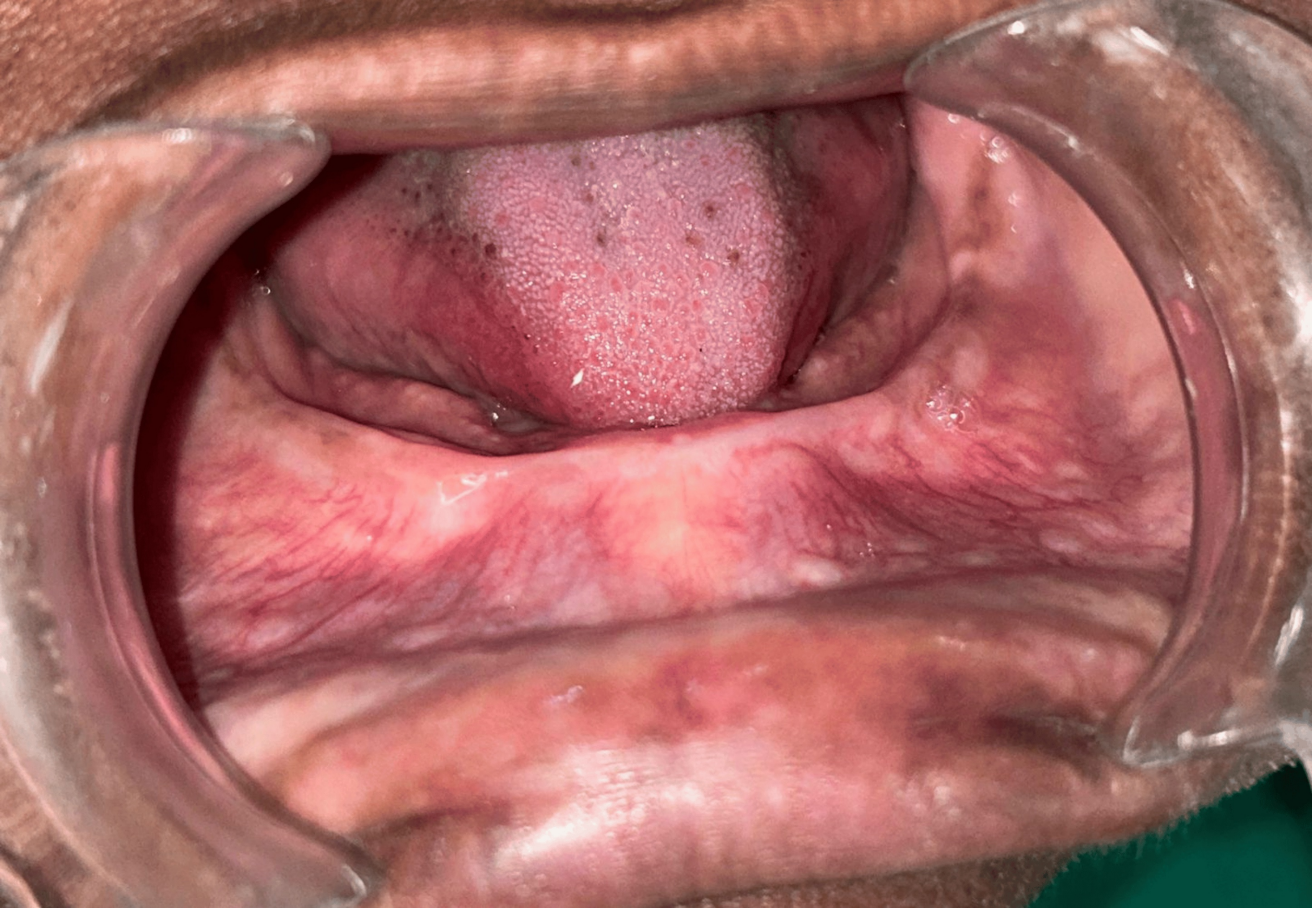
Preoperative intraoral view

A 58-year-old female patient was referred to the Department of Periodontology for vestibular deepening and to increase keratinized attached mucosa to facilitate the adequate fitting of a complete denture. On intraoral examination, a severely resorbed mandible with a low, well-rounded Seibert's Class III ridge with insufficient vestibular depth was observed [5]. The ridge was covered with firm, 3 mm keratinized attached mucosa, with no signs of inflammation or pathology. Insufficient vestibular depth was observed, which posed a critical challenge to denture stability and retention. A vestibuloplasty procedure was planned using the Edlan-Mejchar technique to increase the amount of keratinized attached mucosa and deepen the vestibule. Informed consent was obtained from the patient prior to starting the procedure. Routine blood investigations were carried out, and no abnormalities were detected. The vestibular depth was recorded using a calibrated periodontal probe (UNC-15 probe, Hu-Friedy®, Chicago, Illinois, USA) and found to be approximately 3 mm (Figure 1).

Surgical technique

**Figure 2:**
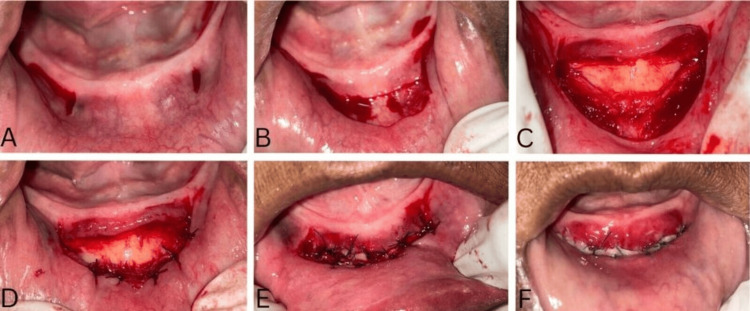
Surgical steps of the Edlan-Mejchar procedure A: Vertical incisions; B: horizontal incisions; C: detachment of the periosteum in the apical direction; D: the periosteum sutured to the lip mucosa; E: mucosal flap sutured at the base of the vestibule; F: seven days postoperative healing view

The preoperative assessment utilized an orthopantomogram to determine the bilateral position of the mental foramen bilaterally. Vertical releasing incisions were given mesial to the mental foramen, extending beyond the mucogingival junction on either side of the desired area where vestibuloplasty was planned (Figure 2A). The vertical incisions were extended apically up to 12 mm into the labial vestibule. The two vertical incisions were joined by a split-thickness horizontal incision involving the epithelium and connective tissue (Figure 2B).

A split-thickness mucosal flap was raised by carefully separating the loose vestibular mucosa from the underlying muscle while maintaining its pedicle attachment to the attached mucosa at the residual ridge. The mucosal flap was reflected superiorly, exposing the periosteum (Figure 2C). A horizontal incision was made below the raised partial-thickness flap, dissecting the periosteum. The periosteum was dissected from the underlying alveolar bone, creating a periosteal flap with its base positioned apically. The upper edge of the periosteal flap was sutured to the mucosal membrane of the lip (Figure 2D). The raised mucosal flap was repositioned inferiorly and secured over the exposed bone using interrupted sutures using vicryl (4-0) suture (Ethicon, Johnson & Johnson, Aurangabad, India), attaching it to the base of the periosteal flap (Figure 2E).

The patient was instructed to use cold fomentation on the first postoperative day and a soft/liquid diet for one week. The patient was also advised not to open her mouth widely during the healing phase. Post-surgical medications prescribed were amoxicillin 500 mg TID for five days and anti-inflammatory (diclofenac 50 mg) BD for five days to control postoperative pain and discomfort. The patient was recalled after one week for follow-up and re-evaluation (Figure 2F).

**Figure 3:**
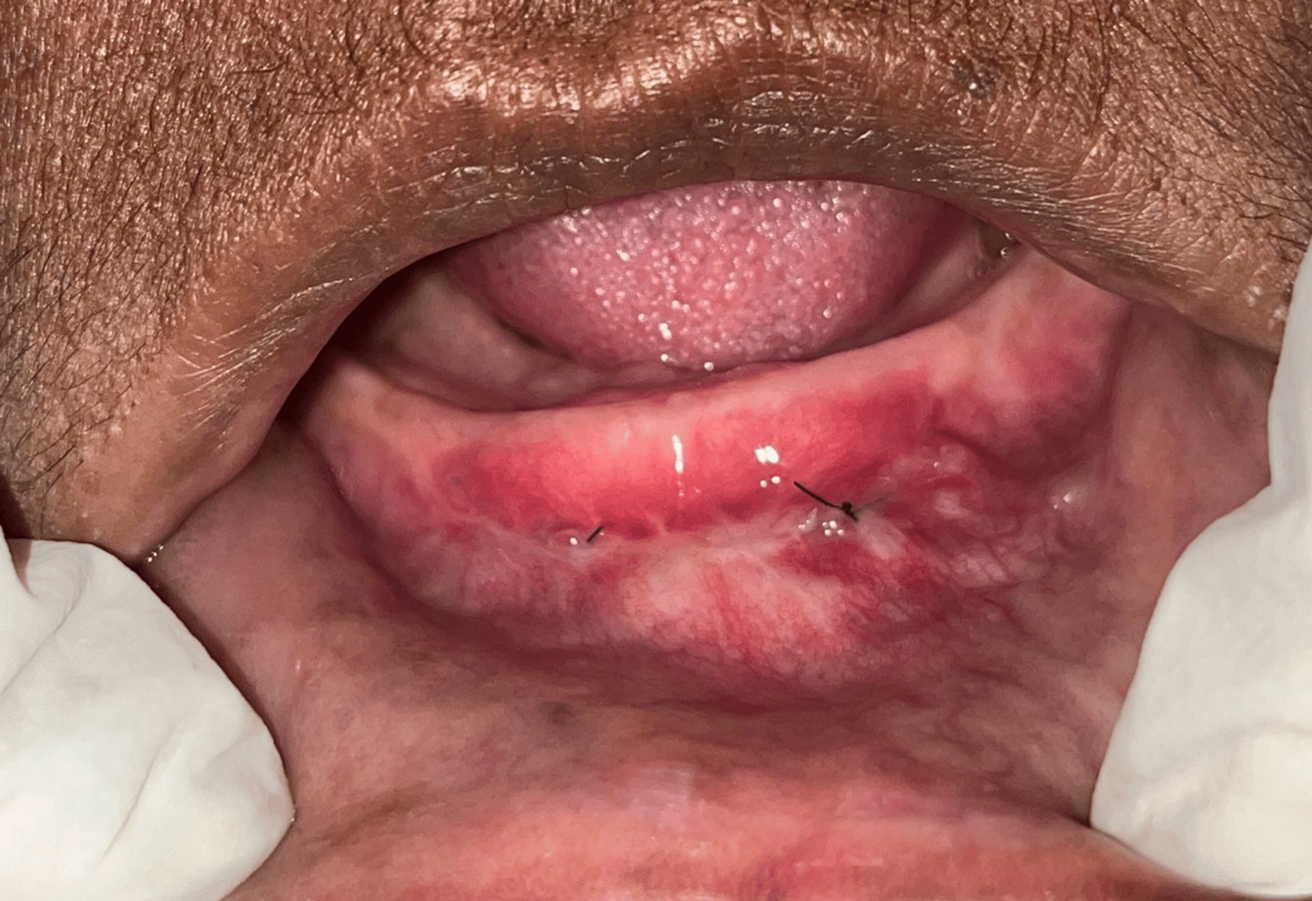
Healing at the one-month follow-up Satisfactory vestibular deepening and increased width of keratinized attached mucosa were achieved.

Figure 3 shows the uneventful healing after one month with satisfactory results. We were able to achieve 6 mm of keratinized attached mucosa and sufficient vestibular depth, which will help in the retention of the complete denture.

Potential complications of the Edlan-Mejchar vestibuloplasty procedure are postoperative pain, speech difficulties, scar formation, wound dehiscence, relapse and loss of vestibular depth, infection risk, postoperative swelling, and oedema. The complications can be minimized by proper case selection, meticulous surgical technique, postoperative care with regular physiotherapy, oral hygiene maintenance, and compliance with follow-up visits.

Table 1 presents the advantages and disadvantages of various vestibuloplasty techniques.

**Table 1:** Advantages and disadvantages of vestibuloplasty techniques

Technique	Advantages	Disadvantages
Clark’s Technique	Simple, suitable for moderate cases	Limited vestibular depth increase
Kazanjian’s Technique	Preserves keratinized mucosa	Risk of relapse, not ideal for severe resorption
Lip Switch	Deepens vestibule effectively	Requires good soft tissue mobility
Edlan-Mejchar	Best for lingual vestibule correction	Post-op healing may be prolonged
Secondary Epithelialization	Good for large defects	Longer healing time, risk of graft failure
Laser-Assisted	Minimal bleeding, faster healing	High cost, expertise required

## Discussion

Alveolar bone resorption following tooth extraction presents significant challenges for dental practitioners, particularly in completely edentulous cases. Pre-prosthetic surgical procedures, especially vestibuloplasty, significantly enhance the treatment outcome with complete removable dentures. Rehabilitating edentulous anterior regions requires attention to both soft tissues and prosthetic components for optimal function and aesthetics.

The Edlan-Mejchar technique effectively addresses soft tissue deficiencies by enhancing the vestibular depth and increasing the width of keratinized mucosa. First described by Edlan and Mejchar (1963), the procedure involves repositioning a split-thickness flap to deepen the vestibule and create a broader zone of keratinized tissue [2]. This technique is particularly useful in edentulous cases, where a shallow vestibule and insufficient keratinized mucosa may lead to challenges with denture retention, patient comfort, and oral hygiene.

Numerous studies support the efficacy of the Edlan-Mejchar procedure in enhancing soft tissue conditions in edentulous patients. Qassadi et al. (2020) demonstrated that vestibuloplasty improves denture stability and comfort by establishing a stable, inflammation-resistant tissue environment [6]. Similarly, Bergström and Halling (1975) reported that vestibuloplasty enhances mucosal support, which in turn contributes to improved prosthetic retention [7].

Several techniques have been described for vestibular extension; however, most techniques have shown limited predictable success in achieving the desired width of the attached gingiva. To date, limited research has evaluated the effectiveness of the Edlan-Mejchar technique. This case report aimed to assess the potential of the Edlan-Mejchar technique in achieving an increase in vestibular depth and to analyze the clinical outcomes at the one-month follow-up. The vestibular deepening achieved through the Edlan-Mejchar technique can be regarded as a modification of the method described by Kazanjian (1924) for deepening the mandibular labial vestibule in edentulous patients [8]. More recent studies, such as those by Wennstrom and Zucchelli (1996), have underlined that adequate keratinized mucosa plays a critical role in maintaining peri-prosthetic tissue health, particularly in edentulous patients [9].

In this case, the Edlan-Mejchar technique was successfully implemented to correct soft tissue deficiencies in the anterior region. The deepened vestibule and enhanced keratinized mucosa will create a stable environment, promoting improved denture retention and facilitating long-term maintenance.

Thoma et al. (2009) have further supported the role of soft tissue augmentation techniques, including the Edlan-Mejchar procedure, in achieving both functional and aesthetic success in edentulous cases [10]. However, as Grunder (2000) noted, the success of these procedures depends heavily on careful case selection, precise surgical technique, and effective collaboration between the periodontist and prosthodontist [11].

Although the outcomes in the present case were positive, certain limitations of the Edlan-Mejchar technique should be acknowledged. These include potential difficulties in patients with systemic conditions that impair healing, the technique-sensitive nature of the surgical procedure, and those needing extensive soft tissue augmentation.

## Conclusions

This case report illustrates the successful use of the Edlan-Mejchar vestibuloplasty technique in a patient with significant mandibular ridge atrophy and limited vestibular depth. Through this procedure, we achieved our goal of increasing the vestibular depth and establishing a 6 mm width of keratinized attached mucosa. The improved vestibular depth is expected to enhance denture stability and function, which will improve patients' overall health.
